# Comparison of the blood, bone marrow, and cerebrospinal fluid metabolomes in children with b-cell acute lymphoblastic leukemia

**DOI:** 10.1038/s41598-021-99147-6

**Published:** 2021-10-04

**Authors:** Jeremy M. Schraw, J. P. Woodhouse, Melanie B. Bernhardt, Olga A. Taylor, Terzah M. Horton, Michael E. Scheurer, M. Fatih Okcu, Karen R. Rabin, Philip J. Lupo, Austin L. Brown

**Affiliations:** 1 Section of Hematology-Oncology, Department of Pediatrics, Baylor College of Medicine, Houston, TX USA; 2 Center for Epidemiology and Population Health, Department of Pediatrics, Baylor College of Medicine, Houston, TX USA; 3 Texas Children’s Cancer and Hematology Centers, Texas Children’s Hospital, Houston, TX USA; 4 Dan L. Duncan Comprehensive Cancer Center, Baylor College of Medicine, Houston, TX USA; 5 Section of Hematology-Oncology, Department of Pediatrics, Baylor College of Medicine, One Baylor Plaza MS: BCM622, Houston, TX 77030 USA

**Keywords:** Cancer epidemiology, Paediatric cancer, Acute lymphocytic leukaemia

## Abstract

Metabolomics may shed light on treatment response in childhood acute lymphoblastic leukemia (ALL), however, most assessments have analyzed bone marrow or cerebrospinal fluid (CSF), which are not collected during all phases of therapy. Blood is collected frequently and with fewer risks, but it is unclear whether findings from marrow or CSF biomarker studies may translate. We profiled end-induction plasma, marrow, and CSF from N = 10 children with B-ALL using liquid chromatography-mass spectrometry. We estimated correlations between plasma and marrow/CSF metabolite abundances detected in ≥ 3 patients using Spearman rank correlation coefficients (*r*_*s*_). Most marrow metabolites were detected in plasma (N = 661; 81%), and we observed moderate-to-strong correlations (median *r*_*s*_ 0.62, interquartile range [IQR] 0.29–0.83). We detected 328 CSF metabolites in plasma (90%); plasma-CSF correlations were weaker (median *r*_*s*_ 0.37, IQR 0.07–0.70). We observed plasma-marrow correlations for metabolites in pathways associated with end-induction residual disease (pyruvate, asparagine) and plasma-CSF correlations for a biomarker of fatigue (gamma-glutamylglutamine). There is considerable overlap between the plasma, marrow, and CSF metabolomes, and we observed strong correlations for biomarkers of clinically relevant phenotypes. Plasma may be suitable for biomarker studies in B-ALL.

## Introduction

Acute lymphoblastic leukemia (ALL) is the most common malignancy diagnosed in children^1,2^. Steady gains in survival have been realized over the last several decades as new agents have been introduced, and 5-year overall survival rates for children treated with modern intensive multi-agent chemotherapy protocols exceed 90%^3,4^. Nonetheless, survival remains dismal (≤ 50%) for some children with high-risk disease^5,6^, and while intensified chemotherapy has resulted in improved survival it has also resulted in high rates of acute toxicities^4,7,8^ (e.g., infections, hepatic and kidney injury, and pancreatitis) and late effects among survivors^9,10^ (e.g., cardiotoxicity, second malignant neoplasms, avascular necrosis, neurocognitive impairment, and frailty). Indeed, in recent trials, further intensification of therapy has either failed to improve survival or produced unacceptable toxicities^7,11,12^. Continued improvements in survival for children with ALL will depend in part on the development of targeted therapies with improved safety profiles, or better methods for identifying and monitoring patients at risk of adverse outcomes. Both will require detailed understanding of host and tumor response to therapy.

Metabolomics encompasses a suite of approaches for systematically identifying and quantifying the small molecules present in a biological system^13^. In the context of ALL, metabolomics has been used to characterize early life environmental exposures associated with ALL incidence^14^, describe differences between cases and healthy controls^15^, predict treatment response^16,17^ and identify factors associated with treatment toxicities^18,19^. Broadly, metabolomics also shows promise for monitoring and understanding drug response^20–23^ and identifying individuals at risk of poor health outcomes^24–27^.

Many metabolomics studies performed in ALL patients have used either supernatant from bone marrow aspirate or cerebrospinal fluid (CSF) from lumbar puncture as the analytic sample^16,18,19^. Bone marrow aspiration and lumbar puncture are invasive and generally require sedation, which has been linked to adverse neurodevelopmental outcomes in children with cancer^28,29^. These procedures are therefore typically performed only at diagnosis for clinical and molecular disease phenotyping, at the end of induction and/or consolidation chemotherapy to assess disease response, and for administration of intrathecal chemotherapy. While metabolomics may be useful for predicting adverse outcomes in children with ALL, it is not clear which samples are best suited to the study of which outcomes, or how venous blood, which can be collected with greater frequency and used to construct longitudinal profiles of response, correlates with bone marrow and CSF. Therefore, we set out to comprehensively describe the correlations between these three matrices. We aim to create a resource that will accelerate ALL research by facilitating translation of findings from bone marrow and CSF.

## Results

### Overview

**Figure 1:**
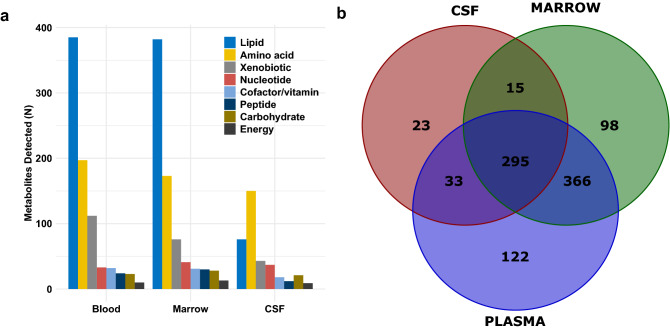
(**a**) Number and class of compounds detected in plasma, bone marrow, and cerebrospinal fluid samples from children with B-ALL. (**b**) Overlap between the plasma, bone marrow, and CSF metabolomes.

Study participants were predominantly male and Latino. Eight patients had National Cancer Institute (NCI) standard-risk disease, and two had high-risk disease. Approximately equal proportions were normal weight and overweight/obese. Median age at diagnosis was 3.5 years (interquartile range [IQR] 2.4–20.6 years) (Table 1). Figure 1 shows the number and class of compounds in plasma, marrow and CSF, as well as the overlap between the three matrices. Plasma demonstrated the greatest richness (N = 816 compounds), followed by marrow (N = 774 compounds) and CSF (N = 366 compounds).

**Table 1:** Demographic and clinical characteristics of B-ALL patients in the study sample.

	N (%)
**Sex**	
Male	7 (70)
Female	3 (30)
**Race/ethnicity**	
Non-Latino White	2 (20)
Latino	8 (80)
**BMI**	
Underweight	1 (10)
Normal weight	5 (50)
Overweight/obese	4 (40)
**Age at diagnosis (years)**	
< 5	7 (70)
5–9	1 (10)
≥ 10	2 (20)
**NCI risk group/treatment protocol**	
Standard risk/AAALL0932	8 (80)
High risk/AALL1131	2 (20)

### Correlations between the plasma and marrow metabolomes

**Figure 2:**
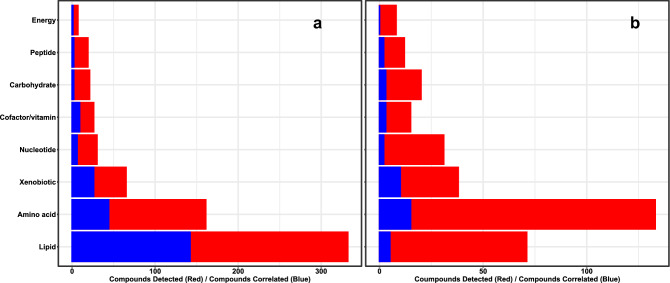
Number of compounds detected (red) and number significantly correlated after 5% false discovery rate adjustment in (**a**) plasma and marrow, and (**b**) plasma and CSF.

Plasma and marrow were similar with respect to the proportion of compounds annotating to different classes, and most marrow compounds were also detected in plasma (N = 661; 81%) (Fig. 1). Overall, we observed moderate-to-strong positive correlations: median *r*_*s*_ was 0.62 (IQR 0.29–0.83), |*r*_*s*_| was > 0.5 for 412 compounds (62.3%) and > 0.75 for 236 compounds (35.7%) (Table 2). After false discovery rate (FDR) adjustment, 35.1% (N = 232) of correlations remained significant (all q < 0.05). Median *r*_*s*_ among these compounds was 0.87 (IQR 0.81–0.92) (Supplementary Table S1 provides data on all metabolites common to the plasma and marrow metabolomes). Amino acids, carbohydrates and peptides were underrepresented in this set, whereas lipids were overrepresented (Table 2) (Fig. 2a). There was evidence that nucleotides were underrepresented among compounds with significant correlations (p = 0.05 by hypergeometric test); 20% (N = 6) were correlated (q < 0.05).

**Table 2:** Number of compounds detected in ≥ 3 plasma and marrow samples, and number that were significantly correlated after FDR correction, by class.

	N detected	Rho, median (IQR)	N correlated^a^	p (under-representation)	p (over-representation)
Total	661	0.62 (0.29–0.83)	207	–	–
Amino acid	161	0.58 (0.32–0.77)	44	0.01	0.99
Carbohydrate	21	0.28 (0.10–0.45)	2	0.01	0.99
Cofactor/vitamin	26	0.59 (0.28–0.82)	9	0.57	0.43
Energy	7	− 0.21 (− 0.36–0.57)	1	0.23	0.77
Lipid	332	0.70 (0.31–0.84)	142	0.99	< 0.001
Nucleotide	30	0.49 (0.32–0.69)	6	0.05	0.95
Peptide	19	0.33 (0.14–0.55)	2	0.02	0.98
Xenobiotic	65	0.77 (0.43–0.90)	26	0.85	0.15

^a^q < 0.05 after Benjamini–Hochberg correction.

We evaluated plasma-marrow correlations for compounds annotated to the Kyoto Encyclopedia of Genes and Genomes (KEGG)^30^ pathway “central carbon metabolism in cancer” (hsa05230), as we had previously reported an association between this pathway and end-induction minimal residual disease (MRD) in diagnostic marrow samples from children with ALL^16^. Two of these compounds, pyruvate (*r*_*s*_ 0.89; q = 0.01) and asparagine (*r*_*s*_ 0.87; q = 0.01), demonstrated significant plasma-marrow correlations.

### Correlations between the plasma and CSF metabolomes

Both the number of compounds detected in CSF and their distribution with respect to class differed relative to marrow and plasma. In particular, we observed proportionately fewer lipids and proportionately more amino acids (Fig. 1a). Similar to marrow, the majority of compounds in CSF were also detected in blood (N = 328, 89.6%) (Fig. 1b). Plasma-CSF correlations were weaker than plasma-marrow correlations on average (median *r*_*s*_ 0.37, IQR 0.07–0.70) (Table 3). We observed moderate correlations (0.5 <|*r*_*s*_|< 0.75) for 63 compounds (19.2%), and strong correlations (|*r*_*s*_|≥ 0.75) for 65 compounds (19.8%). Forty compounds (12.2%) had q-values < 0.05, and these demonstrated strong positive correlations on average (median *r*_*s*_ 0.93, IQR 0.85–0.95) (Supplementary Table S2 provides data on all metabolites common to the plasma and CSF metabolomes). Xenobiotics were overrepresented among compounds with statistically significant FDR-adjusted correlations, whereas there was some evidence that lipids were underrepresented (p = 0.09) (Table 3) (Fig. 2b).

**Table 3:** Number of compounds detected in ≥ 3 plasma and CSF samples, and number that were significantly correlated after FDR correction, by class.

	N detected	Rho, median (IQR)	N correlated^a^	p (under-representation)	p (over-representation)
Total	328	0.37 (0.07–0.70)	40	–	–
Amino acid	133	0.45 (0.13–0.72)	15	0.41	0.59
Carbohydrate	20	0.15 (− 0.08–0.57)	3	0.79	0.22
Cofactor/vitamin	15	0.48 (0.11–0.74)	3	0.91	0.10
Energy	8	0.17 (0.08–0.31)	0	0.35	0.65
Lipid	71	0.28 (− 0.04–0.57)	5	0.09	0.91
Nucleotide	31	0.31 (0.16—0.46)	2	0.24	0.76
Peptide	12	0.13 (− 0.28–0.37)	2	0.83	0.17
Xenobiotic	38	0.76 (0.28–0.94)	10	0.99	0.002

^a^q < 0.05 after Benjamini–Hochberg correction.

We previously reported associations between CSF abundances of asparagine, dimethylglycine and gamma-glutamylglutamine and cancer-related fatigue in children with ALL^18^. Here, we observed significant plasma-CSF correlations for dimethylglycine (*r*_*s*_ 0.84; q = 0.04) and gamma-glutamylglutamine (*r*_*s*_ − 0.93; q = 0.002). Interestingly, the inverse correlation between CSF and plasma abundances of gamma-glutamylglutamine was the strongest observed, and one of only two which were statistically significant.

## Discussion

Overall, we found that plasma metabolomics is appropriate for estimating metabolic processes in the marrow or CSF of children with B-ALL. The majority of compounds detected in marrow or CSF were also detected in plasma and (especially for marrow) the number and type of compounds was similar. Substantial proportions (62% in marrow and 41% in CSF) demonstrated moderate or strong correlations with plasma and many (35% in marrow, 16% in CSF) remained statistically significant at q < 0.05 after multiple testing correction. Of note, we reported strong correlations for compounds previously associated with cancer-related fatigue^18^ and MRD^16^, supporting that blood may be useful for biomarker studies of these compounds and endpoints. These findings have practical implications for future metabolomics studies, since blood can be collected more frequently and less invasively.

When evaluating plasma and bone marrow, we observed a robust correlation for asparagine. Asparaginase has long been utilized in ALL chemotherapy, following the discovery of its anti-leukemic effect, mediated by serum asparagine and glutamine depletion, and clinical trials demonstrating improved survival for asparaginase-treated patients^31,32^. In contrast to a previous magnetic resonance spectroscopy (MRS) and gas chromatography-mass spectrometry (GC–MS)-based untargeted metabolomics study which reported total depletion of asparagine at D29 in patients who received PEG-asparaginase between induction D4 and D6^17^, we detected asparagine in plasma and marrow samples from all children. Patients on both induction protocols (AALL0932 and AALL1131) received PEG-asparaginase on induction D4^12,33^; our observation is consistent that of Angiolillo et al., who demonstrated that asparagine levels begin to recover 20–25 days after PEG-asparaginase administration^34^. Given the importance of asparagine depletion in ALL chemotherapy, it may be noteworthy that it was readily detected in plasma using this approach, and that plasma and marrow asparagine abundances correlated strongly. Conversely, plasma and CSF asparagine abundances were not significantly correlated. Given that CSF asparagine depletion is also essential, measurement of plasma asparagine alone may be inadequate to quantify the extent and duration of asparagine depletion in the CSF^35^.

We also observed a strong positive correlation between marrow and plasma pyruvate abundances. In a study of children with newly diagnosed ALL we reported that bone marrow pyruvate abundance at diagnosis was associated with subsequent MRD (1.9-fold increase among MRD-positive patients, p = 0.02)^16^. Pyruvate is a key intermediate in glycolysis and gluconeogenesis. Altered glucose metabolism has been described in ALL cells^36,37^, and we and others have demonstrated that inhibitors of glycolysis exert anti-leukemic effects in vitro^16,36^. Whether plasma pyruvate abundances similarly associate with treatment response is unclear, but is a promising area for future research that aims to leverage metabolomics to understand ALL outcomes.

In our analysis of plasma and CSF, we observed a strong, albeit inverse, correlation between plasma and CSF abundances of gamma-glutamylglutamine. We previously reported an inverse (cross-sectional) association of CSF gamma-glutamylglutamine abundance and fatigue scores during post-induction chemotherapy, and reported that its abundance at the time of diagnosis was inversely correlated with fatigue severity at the start of delayed intensification^18^. Gamma-glutamylglutamine is an intermediate in the gamma-glutamyl cycle, in which gamma-glutamyl amino acids are formed by the transfer of glutamyl moieties (e.g., from glutathione to glutamate), then subsequently cleaved to the free amino acid and 5-oxoproline. This pathway may play a role in the regulation of amino acid transport across the blood–brain barrier^38^, which could explain the observation that plasma and CSF gamma-glutamylglutamine abundances were inversely correlated.

Saito et al. investigated changes in the plasma metabolome of patients with ALL pre- and post-induction and observed that > 20% of compounds, most notably lipids, were altered^39^. The authors hypothesized that these alterations may affect the risk of adverse events in children with ALL, as previous studies suggest effects of docosahexaenoic acid (DHA)^40–42^ and phosphatidylethanolamines^43^ on asparaginase-associated pancreatitis and relapse. We found that lipids demonstrated somewhat stronger plasma-marrow correlations than other compounds.

Tiziani et al. compared plasma and bone marrow samples from N = 10 children with ALL at diagnosis and reported differences among amino acids and ketones^17^. Collectively, these findings suggest that the plasma and marrow lipids are well correlated, but that special care may be required when comparing diagnostic samples due to the high titer of leukemic cells. These findings may have particular implications for investigators wishing to perform longitudinal assessments or utilize lipidomics approaches.

Our study should be interpreted in light of certain strengths and limitations. Our sample size was small, which limited our ability to detect statistically significant correlations and prevented us from performing analyses stratified by factors such as sex, age at diagnosis, BMI or end-induction MRD status. The study was also cross-sectional, with all samples collected at the end of induction chemotherapy. On the other hand, we performed metabolomic profiling using a well-described untargeted platform with broad coverage and an extensive reference panel, which identified > 1000 unique features. Because this platform is semi-quantitative, we present data on relative abundances rather than absolute concentrations. In future studies, targeted approaches may allow for improved quantitation of metabolite or drug concentrations. Finally, the study sample was relatively homogenous, consisting entirely of pediatric patients with newly diagnosed B-ALL treated on standard protocols. This likely reduced heterogeneity in our analysis, but we caution that it is unknown whether our findings may be applicable to T-ALL.

## Conclusions

Our findings highlight that untargeted plasma metabolomics readily detects compounds associated with the clinically relevant phenotypes of end-induction MRD and fatigue in children with ALL. ALL metabolomics is a nascent field and many of the studies performed to date have used bone marrow or CSF, which may be limiting for broader applications. To accelerate translation of these findings, we have comprehensively described the correlations between the blood, marrow, and CSF metabolomes at end-induction (see Supplementary Material for correlations for all detected compounds). We observed generally strong correlations between plasma and marrow, suggesting that plasma may be an optimal and readily available source for use in future studies. In particular, quantitative evaluations of candidate biomarkers and longitudinal assessments of the plasma metabolome across therapy may be informative for precision medicine approaches, and ultimately drive improvements in survival for children with ALL. While we highlight findings for putative biomarkers of MRD and fatigue, we note that metabolomics has scarcely been applied to study other important outcomes such as cardiotoxicity, hepatotoxicity, and relapse, all of which may be promising directions for future research.

## Methods

### Study population

Participants (N = 10) were children diagnosed with B-lineage ALL in 2017–2018, and treated at Texas Children’s Hospital on or according to Children’s Oncology Group protocols appropriate for their age and disease characteristics. We obtained plasma, bone marrow and cerebrospinal fluid samples at the end of induction chemotherapy, during routine clinical care. We extracted demographic and clinical data including sex, age, race/ethnicity, height, weight, disease type, NCI risk group and end-induction MRD status from the electronic health record. We defined children as overweight if their body mass index (BMI) was ≥ 85th percentile for their age and sex, and as obese if it was ≥ 95th percentile. We defined children as NCI standard risk if they were < 10 years of age at diagnosis and had an initial white blood cell count of < 50,000/µL and high risk otherwise^44^. Children whose end-induction marrow specimen contained ≥ 0.01% leukemic blasts, measured by flow cytometry, were considered MRD-positive. This study was approved by the Baylor College of Medicine Institutional Review Board (H-29892) and performed in accordance with the Declaration of Helsinki. Informed consent was obtained from the parents/guardians of all participating children. All procedures were performed in accordance with the relevant guidelines and regulations.

### Metabolomic profiling

Plasma, marrow and CSF samples were processed according to standard methods and stored at – 80 °C until they were batch shipped to Metabolon Inc. (Morrisville, NC) for analysis using the Precision Metabolomics™ platform. Sample processing and analysis procedures for this platform have been described previously^16,45^. Briefly, methanol was added and samples were centrifuged to precipitate proteins. The resulting supernatant was divided into four extracts: two were analyzed by reverse phase ultrahigh performance liquid chromatography-tandem mass spectrometry (UPLC-MS/MS) methods with positive ion mode electrospray ionization, one by UPLC-MS/MS with negative ion mode electrospray ionization, and one by hydrophilic interaction UPLC-MS/MS. Organic solvent was removed using a TurboVap® (Zymark) and samples were stored overnight under nitrogen prior to analysis. Extracts were dried, reconstituted and analyzed using untargeted, UPLC-MS/MS-based approaches on a Waters ACQUITY chromatograph (Waters, Milford, MA) and a Thermo Scientific Q-Exactive spectrometer (Thermo Fisher Scientific, Waltham, MA). Instrument variability was measured by calculating the median relative standard deviation (RSD) for internal standards added to each sample prior to analysis, and was 4% for plasma and CSF and 5% for marrow. In addition to internal standards, a small amount of each sample was pooled and used as technical replicates throughout. Total process variability (6% for plasma, 7% for marrow and 9% for CSF) was determined by calculating the median RSD for all endogenous metabolites present in these pooled matrix samples. Data extraction, peak identification and compound identification were performed by Metabolon using an in-house bioinformatics pipeline. Peaks were quantified using area under the curve. Values were normalized to total protein content, measured by the Bradford assay, and log-transformed. Compounds were identified by comparing retention index and spectral data against a library of > 3000 commercially available purified standards and categorized as belonging to one of the following classes: amino acid, carbohydrate, cofactor/vitamin, energy, lipid, nucleotide, peptide or xenobiotic.

### Statistical methods

Compounds were level scaled using the median prior to analysis^46^. For endogenous compounds (amino acids, carbohydrates, cofactors/vitamins, lipids, nucleotides and peptides) we imputed missing values with half the minimum observed value^47^, as it was assumed that these were present in samples below the limit of detection. Missing values were not imputed for xenobiotics. We computed non-parametric Spearman rank correlation coefficients (*r*_*s*_) to describe correlations of compound abundances between plasma-marrow and plasma-CSF. We applied the Benjamini–Hochberg correction to maintain a 5% FDR, defining q < 0.05 as the threshold for statistical significance. We visualized these correlations using heatmaps and summarized our results using median and IQR. To determine whether compounds from certain classes were overrepresented among those with FDR-significant correlations, we used hypergeometric tests, with p < 0.05 as the threshold for statistical significance^48^. We excluded compounds detected in fewer than three children from all analyses. Analyses were performed in R v3.6.3 (R Foundation, Vienna, Austria).

## Supplementary Information

Supplementary Information.

## Data Availability

Underlying data are publically available through Mendeley Data (DOI: 10.17632/xz4m36dgzh.2).
